# Supplementary material for: How ice grows from premelting films and water droplets
Source: Nat Commun. 2021 Jan 11;12:239. doi: 10.1038/s41467-020-20318-6 (PMC7801427; doi:10.1038/s41467-020-20318-6)
Supplement: Supplementary file 1 — Supplementary Information [file 41467_2020_20318_MOESM1_ESM.pdf]

How ice grows from premelting film and water droplets (Supplementary Information)

David N. Sibley[1], Pablo Llombart[2,3], Eva G. Noya[2], Andrew J. Archer[1] and Luis G. MacDowell[3,*]

[1]: Department of Mathematical Sciences, Loughborough University, Loughborough LE11 3TU, United Kingdom.

[2]: Instituto de Química Física Rocasolano, CSIC, Calle Serrano 119, 28006 Madrid, Spain.

[3]: Departamento de Química Física (Unidad de I+D+i Asociada al CSIC), Facultad de Ciencias Químicas, Universidad Complutense de Madrid, 28040, Spain.

SUPPLEMENTARY FIGURE 1

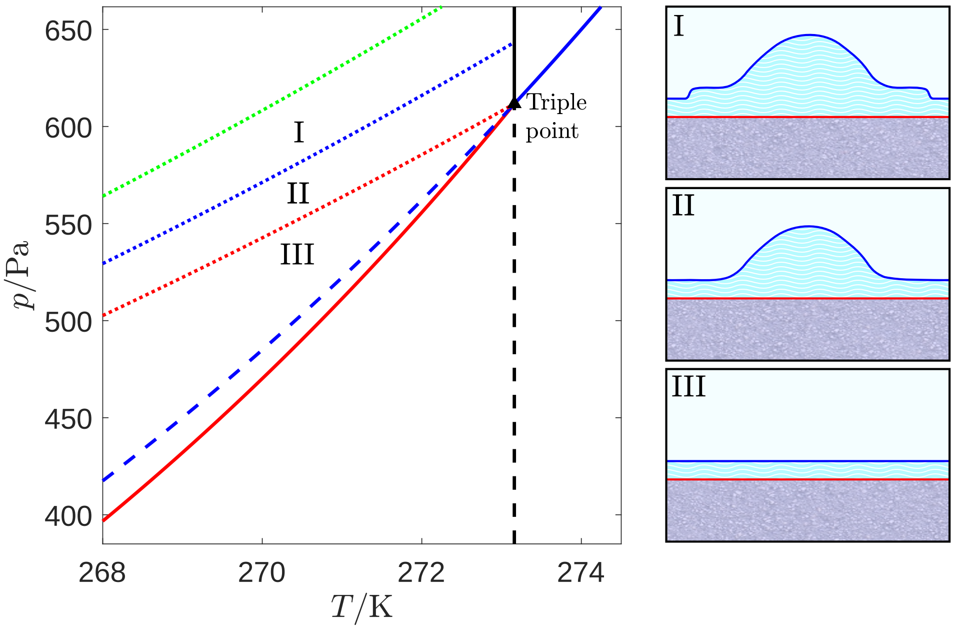

Supplementary Fig. 1: **Kinetic wetting phase diagram for the ice/vapor surface with linear growth ($u = 0$).** On the left is the equilibrium phase diagram of water in the neighborhood of the triple point. The solid lines are the melting (black), vaporization (blue) and sublimation (red) lines. The dashed lines are metastable prolongations of the melting and vaporization lines. Dotted red and blue lines are kinetic transition lines which describe the transitions observed in experiments between the states illustrated on the right, namely (I) a spreading film below a droplet, (II) a droplet on top of a homogeneous surface and (III) a homogeneous surface [1]. The green dotted line is the kinetic spinodal line where quasi-stationary states are no longer stable. The kinetic transition lines shown here have been calculated using the model in Eqs. (3) and (4) of the main text, assuming linear growth ($w = 0$). The interface potential is scaled by a factor of 30 in order to illustrate how the separation between kinetic phase lines increases as the depth of the minima in the interface potential increase (See Fig. 3 of the main text with the associated discussion and Supplementary Note 3 below).

SUPPLEMENTARY FIGURE 2

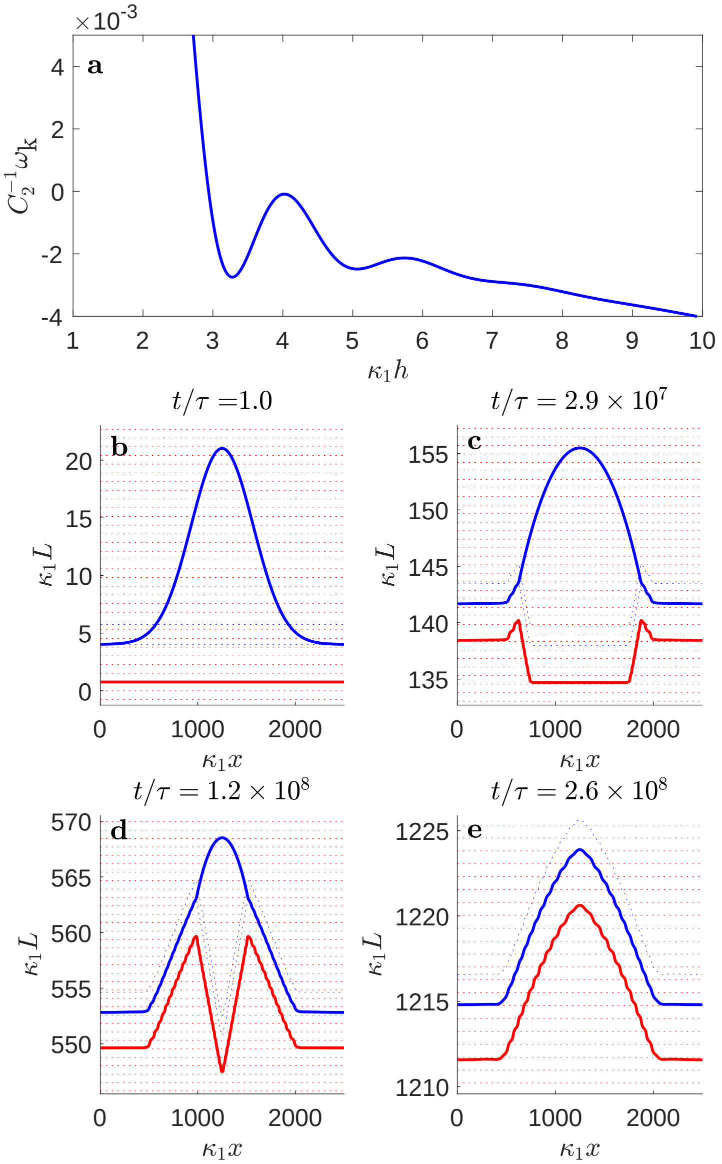

Supplementary Fig. 2: **Surface dynamics for an initial droplet at a state point above the kinetic liquid-vapor coexistence line**, (p, T) =(517.5 Pa, 269.5 K), corresponding to the triangle (△) symbol in Fig. 3 (main text). The color/style code of the lines is as explained in captions to Fig. 4 (main text). Compared to Fig. 4 (**f-j**) (main text) for the dynamics of the same droplet right below the kinetic liquid-vapor coexistence, the droplet is now stabilized for a long period, and a crater is formed below.

SUPPLEMENTARY FIGURE 3

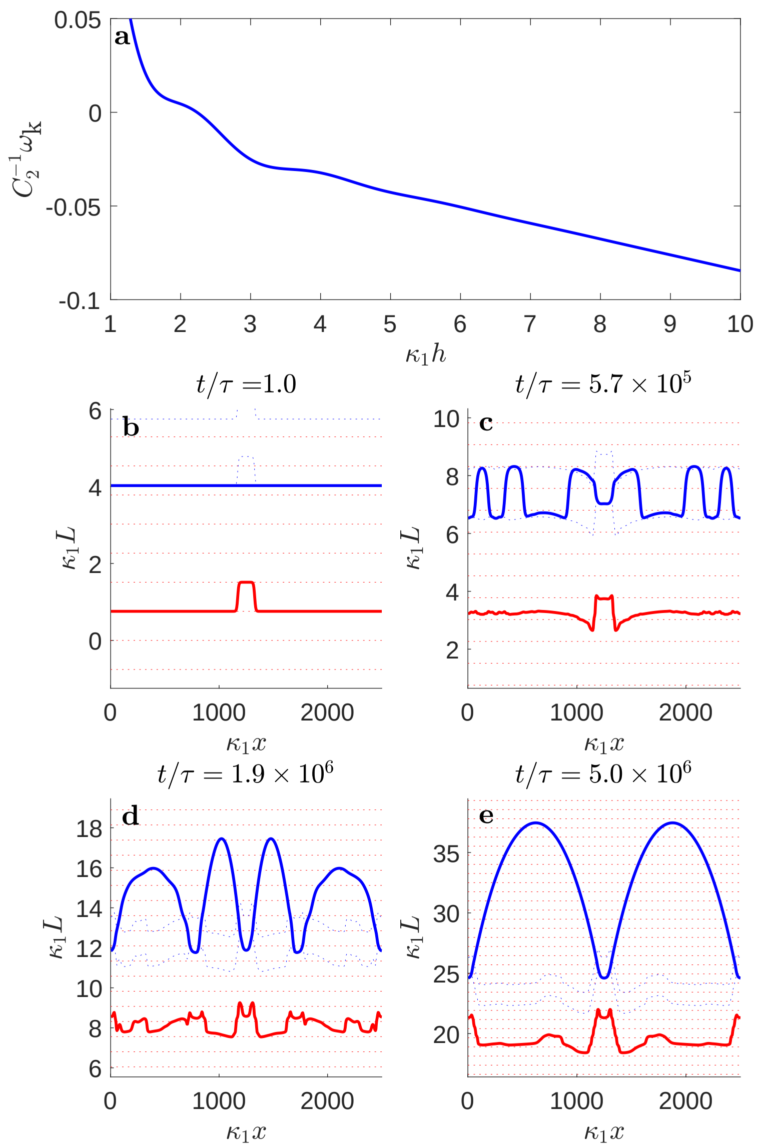

Supplementary Fig. 3: **Surface dynamics for a system with an initial terrace at a state point above the kinetic spinodal line**, (p, T) =(535 Pa, 269.5 K), corresponding to the lozenge (◊) symbol in Fig. 3 (main text). The color/style code of the lines is as explained in captions to Fig. 4 (main text). Compared to Fig. 4 (**b-e**) (main text) for the dynamics of the same initial terrace, the flat liquid-vapor surface becomes unstable and forms satellite droplets that grow and aggregate over time to leave the ice surface covered in a thick film of liquid.

SUPPLEMENTARY TABLE 1

Property	Value
C_1	3.143×10^{-3} J m^{-2}
C_2	4.116×10^{-2} J m^{-2}
κ_1	2.043×10^{9} m^{-1}
q_0	7.148×10^{9} m^{-1}
α	5.144
B	7.875×10^{-31} Jm
f	1.106 (unitless)
a	3.03×10^{7} m^{-1}
b	5.0×10^{8} m^{-1}
α-minimum	1.6 nm
β-minimum	2.4 nm
Π^*, $\alpha - \beta$ transition	-4.60×10^{4} Pa
Π^*, β spinodal	-1.02×10^{5} Pa

Supplementary Table 1: **Parameters used in the interface potential**, $g(h)$ with details on the locations of the α and β minima and spinodals. Further details of the fitting procedure may be found in Supplementary Note 3 below.

SUPPLEMENTARY TABLE 2

Property	Value	Reference
T_{t}	273.16 K	[2]
T_{t}	0.1 °C	[2]
p_{t}	611.65 Pa	[2]
ρ_{l}	55 498 mol m^{-3}	[2]
ρ_{s}	50 888 mol m^{-3}	[3]
ρ_{v}	0.2694 mol m^{-3}	[2]
ΔH_{sv}	51 059 J mol^{-1}	[4]
ΔH_{lv}	45 051 J mol^{-1}	[4]
ΔH_{sl}	6 008 J mol^{-1}	[4]

Supplementary Table 2: **Triple point data of water**. These results are used for the calculation of thermodynamic functions described in the Supplementary Note 6. Conversion from mass to molar units performed assuming $M_{\mathrm{w}} = 18.015$ g/mol.

SUPPLEMENTARY TABLE 3

Property	Value	Source
d_{B}	0.37×10^{-9} m	[5]
ρ_{lv}	$p_{\mathrm{lv}}/(R_{\mathrm{c}}T)$	ideal gas law
T_{c}	$T - 273.15$ °C	Celsius scale
ρ_{l}	$55502 + 3.4549T_{\mathrm{c}} - 0.44461T_{\mathrm{c}}^2 \ldots$ $\ldots + 0.0028885T_{\mathrm{c}}^3 - 0.00031898T_{\mathrm{c}}^4$ mol m^{-3}	[6, 7]
ρ_{s}	$50885 - 9.71T_{\mathrm{c}} - 0.03T_{\mathrm{c}}^2$ mol m^{-3}	[5]
γ_{sl}	$(28 + 0.25T_{\mathrm{c}}) \times 10^{-3}$ J m^{-2}	[5]
γ_{lv}	$(75.7 - 0.1775T_{\mathrm{c}}) \times 10^{-3}$ J m^{-2}	[8]
η	$1.39 \times 10^{-4}(T/225 - 1)^{-1.64}$ kg m^{-1} s^{-1}	[9]
u	1.3×10^{-4} J m^{-2}	This work
k_{lv}	$3.4 \times 10^{-10}\rho_{\mathrm{lv}}T^{-1/2} \times 10^{-3}$ m s^{-1} Pa^{-1}	Knudsen-Hertz law
k_{sl}	$6.4k_{\mathrm{lv}}$	Slope of phase line

Supplementary Table 3: **Temperature dependent coefficients for use in the mesoscopic calculations**. T refers to absolute temperature in K. T_c refers to temperature in the Celsius scale. Further details on the derivation of these coefficients may be found in Supplementary Note 7 below.

SUPPLEMENTARY NOTE 1: NUMERICAL CALCULATION OF THE INTERFACE POTENTIAL

Definition of the interface potential

The excess grand potential Ω (Landau free energy) per unit area for a liquid film of thickness h on a planar solid surface in equilibrium with a bulk vapor phase with chemical potential μ and temperature T is

$$\frac{\Omega + p_{\rm v}V}{A} = \gamma_{\rm sl} + \gamma_{\rm lv} + g(h;T) - \Delta p_{\rm lv}(T,\mu)h \tag{1}$$

$$\equiv \gamma_{\rm sl} + \gamma_{\rm lv} + \omega(h;T,\mu), \tag{2}$$

where V is the volume of the system, A is the area of the surface, $\gamma_{\rm sl}$ is the solid/liquid interfacial tension, $\gamma_{\rm lv}$ is the liquid/vapor interfacial tension, $g(h)$ is the interface potential for the film at liquid-vapor coexistence, often referred to as the binding potential, and $\Delta p_{\rm lv}(T,\mu) = p_{\rm l}(T,\mu) - p_{\rm v}(T,\mu)$ is the pressure difference of the bulk liquid and vapor phases at the chemical potential of the bulk vapor. The potential $\omega(h;T,\mu)$ is the effective interface potential that determines the interfacial phase behavior.

In relevant previous work, the interface potential of liquid films adsorbed on an inert substrate was calculated by performing grand-canonical simulations at liquid-vapor coexistence [10, 11]. In that case, $\omega(h;T,\mu) = g(h;T)$, and the free energy may be evaluated from $A\,\omega(h;T,\mu) = -k_{\rm B}T\ln P(h)$, where $P(h)$ is the probability distribution of h, collected during the grand canonical simulation with enhanced sampling techniques and $k_{\rm B}$ is Boltzmann's constant. Effectively, the procedure is equivalent to performing a series of canonical simulations at different film thicknesses [12].

For the case of a one component system with liquid adsorbed at the solid/vapor interface, the above method cannot be applied, because the three phase system at fixed temperature only exists at equilibrium at the solid/vapor coexistence chemical potential. Instead, we perform a set of fixed-NVT simulations at different temperatures (N is the number of molecules), similar to previous calculations in studies of the interface potential for grain boundary premelting [13, 14].

For a liquid film adsorbed at the solid/vapor interface along the sublimation line $(T,\mu_{\rm sv}(T))$, Supplementary Eq. (1) gives

$$\omega(h;T,\mu_{\rm sv}) = g(h;T) - \Delta p_{\rm lv}(T)|_{\rm sv}h, \tag{3}$$

where $g(h;T)$ is the interface free energy for the film along the liquid-vapor coexistence line, and $\Delta p_{\rm lv}(T)|_{\rm sv} = p_{\rm l}(T,\mu_{\rm sv}) - p_{\rm v}(T,\mu_{\rm sv})$ is the pressure difference between liquid and vapor bulk phases at the solid-vapor coexistence chemical potential.

Performing simulations of the solid phase at constant temperature, initiated in a vacuum, the system equilibrates into a state of solid/vapor coexistence, with a premelting liquid film at the interface with thickness dictated by imposed thermodynamic conditions. At this temperature, the film thickness fluctuates according to a probability distribution $P(h;T,\mu_{\rm sv})$, which can easily be collected during the course of the simulation, as shown in Supplementary Fig. 4.

The interface potential in the range of observed film thicknesses may be calculated as

$$g(h;T) = -\frac{k_BT}{A}\ln P(h;T,\mu_{\rm sv}) + \Delta p_{\rm lv}(T)|_{\rm sv}h + C_T, \tag{4}$$

where C_T is an arbitrary constant. By performing a sequence of simulations at different temperatures, one obtains a set of piecewise potentials $g(h;T_i)$, which overlap for small ranges of h, provided the simulations are performed at sufficiently close temperature intervals. The right hand panel of Supplementary Fig. 4 shows the set of piecewise functions obtained at a series of different temperatures, with values as indicated in the key. Since the temperature dependence of $g(h;T)$ is small, the piecewise function can be combined into a single continuous interface potential by choosing suitable constants C_{T_i}. The resulting function is continuous and shows no apparent singularities, consistent with the assumption of weak temperature dependence of the various piecewise terms $g(h;T_i)$.

Calculation of the pressure difference $\Delta p_{\rm lv}(T)|_{\rm sv}$

In order to evaluate the interface potential, we must first determine $\Delta p_{\rm lv}(T)|_{\rm sv}$. We start from the Gibbs-Duhem thermodynamic relation

$$Nd\mu = -SdT + Vdp, \tag{5}$$

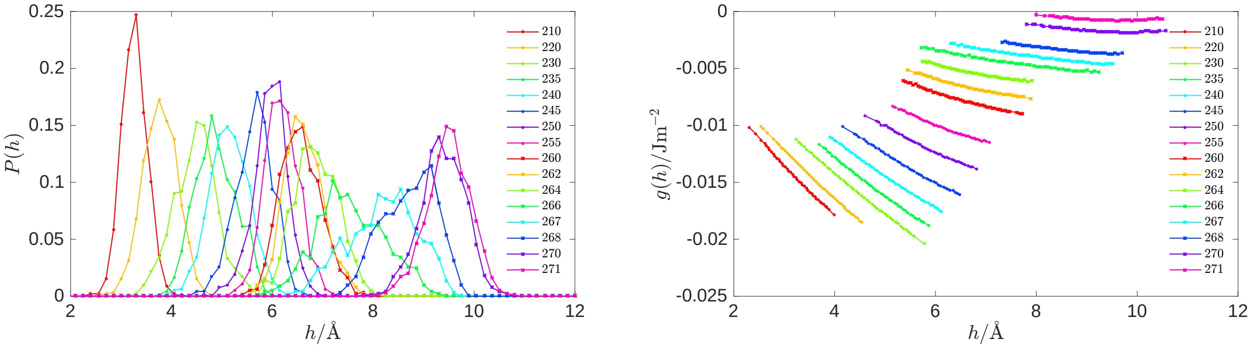

Supplementary Fig. 4: Left: The global film height probability distribution, obtained from a sequence of independent simulations at fixed NVT and for a range of different temperatures (210-271 K), as given in the key. Right: The corresponding piecewise interface potentials.

where S is the entropy. From this we obtain the following equivalent pair of relations

$$d\mu = -sdT + \frac{1}{\rho}dp, \tag{6}$$

$$dp = \rho s dT + \rho d\mu, \tag{7}$$

where $s = S/N$ is the entropy per particle and $\rho = N/V$ is the number density. At phase coexistence, μ, p and T are equal in the two coexisting phases. Hence, along the the solid (subscript s) and vapor (v) coexistence line we have $d\mu_{\rm s} = d\mu_{\rm v}$, $dp_{\rm s} = dp_{\rm v}$ and $dT_{\rm s} = dT_{\rm v}$. Therefore, from the first of these together with Supplementary Eq. (6) we obtain the familiar Clausius-Clapeyron equation for the variation of the vapor pressure along the sublimation line

$$\left.\frac{dp}{dT}\right|_{\rm sv} = \rho_s\rho_{\rm v}\frac{s_{\rm s}-s_{\rm v}}{\rho_{\rm v}-\rho_{\rm s}}. \tag{8}$$

Similarly, from Supplementary Eq. (7) we obtain

$$\left.\frac{d\mu}{dT}\right|_{\rm sv} = \frac{\rho_{\rm v}s_{\rm v}-\rho_{\rm s}s_{\rm s}}{\rho_{\rm s}-\rho_{\rm v}}. \tag{9}$$

Thus, from Supplementary Eq. (8) the variation of vapor pressure along the sublimation line is

$$dp_{\rm v}|_{\rm sv} = \rho_s\rho_{\rm v}\frac{s_{\rm s}-s_{\rm v}}{\rho_{\rm v}-\rho_{\rm s}}dT, \tag{10}$$

whereas the pressure variations of the liquid phase is given more generally by Supplementary Eq. (7) as

$$dp_{\rm l} = \rho_{\rm l}s_{\rm l}dT + \rho_{\rm l}d\mu. \tag{11}$$

However, we must evaluate the liquid pressure along the sublimation line, so μ is not an independent variable. Rather, it is given by the Clausius-Clapeyron type Supplementary Eq. (9), and thus

$$dp_{\rm l}|_{\rm sv} = \rho_{\rm l}s_{\rm l}dT + \rho_{\rm l}\frac{\rho_{\rm v}s_{\rm v}-\rho_{\rm s}s_{\rm s}}{\rho_{\rm s}-\rho_{\rm v}}dT. \tag{12}$$

Therefore, the variation of $d(p_{\rm l}-p_{\rm v})|_{\rm sv}$ along the sublimation line is obtained from Supplementary Eqs. (10) and (12) after some rearrangements, as

$$d(p_{\rm l}-p_{\rm v})|_{\rm sv} = \left.\frac{\rho_{\rm s}\rho_{\rm l}s_{\rm l}-\rho_{\rm v}\rho_{\rm l}s_{\rm l}+\rho_{\rm l}\rho_{\rm v}s_{\rm v}-\rho_{\rm l}\rho_{\rm s}s_{\rm s}+\rho_{\rm s}\rho_{\rm v}s_{\rm s}-\rho_{\rm s}\rho_{\rm v}s_{\rm v}}{\rho_{\rm s}-\rho_{\rm v}}\right|_{\rm sv} dT. \tag{13}$$

In principle, this equation could be integrated starting from the triple point, where $p_{\rm l} - p_{\rm v} = 0$, down to lower temperatures, by using experimental or simulation data for entropies and densities along the sublimation line. A zeroth order integrated form of this equation may be found in Elbaum and Schick [15].

Here, we take a different more convenient approach by expressing this equation in terms of liquid-vapor and solid-vapor coexistence pressures, which are known from experiments with great accuracy. To achieve this, we first notice $\rho_{\rm v} \ll \rho_{\rm l}$ and $\rho_{\rm v} \ll \rho_{\rm s}$. Therefore, the exact result in Supplementary Eq. (13) can be greatly simplified with only a very small loss in accuracy, to

$$d(p_{\rm l} - p_{\rm v})|_{\rm sv} = -\rho_{\rm l}(s_{\rm s} - s_{\rm l})dT|_{\rm sv}. \tag{14}$$

Now, we write

$$(s_{\rm s} - s_{\rm l})|_{\rm sv} = \left[(s_{\rm s} - s_{\rm v}) - (s_{\rm l} - s_{\rm v})\right]_{\rm sv}. \tag{15}$$

Furthermore, assuming the vapor behaves as an ideal gas so that (i) $s = -k_{\rm B}(\ln(\Lambda^3\rho) - 1)$, where Λ is the thermal de Broglie wavelength, and (ii) $\rho = p/k_{\rm B}T$, we can write the vapor entropy at the sublimation line in terms of the vapor entropy at the condensation line as

$$s_{\rm v}|_{\rm sv} = s_{\rm v}|_{\rm lv} + k_{\rm B}\ln\frac{p_{\rm lv}}{p_{\rm sv}}. \tag{16}$$

Substituting this into Supplementary Eq. (15) and noting that the entropy of the incompressible liquid phase hardly changes at all, which means that we may approximate $s_{\rm l}|_{\rm sv} = s_{\rm l}|_{\rm lv}$, so that from Supplementary Eq. (15) and Supplementary Eq. (16) we can write

$$(s_{\rm s} - s_{\rm l})|_{\rm sv} = (s_{\rm s} - s_{\rm v})|_{\rm sv} - (s_{\rm l} - s_{\rm v})|_{\rm lv} + k_{\rm B}\ln\frac{p_{\rm lv}}{p_{\rm sv}}. \tag{17}$$

Substituting this into Supplementary Eq. (14) then yields:

$$d(p_{\rm l} - p_{\rm v})|_{\rm sv} = -\rho_{\rm l}\left[(s_{\rm s} - s_{\rm v})|_{\rm sv} - (s_{\rm l} - s_{\rm v})|_{\rm lv} + k_{\rm B}\ln\frac{p_{\rm lv}}{p_{\rm sv}}\right]dT, \tag{18}$$

where now both $(s_{\rm s} - s_{\rm v})|_{\rm sv}$ and $(s_{\rm l} - s_{\rm v})|_{\rm lv}$ are actual entropies of phase change. Invoking the Clausius-Clapeyron Supplementary Eq. (8) for these two quantities, assuming $\rho_{\rm v} \ll \rho_{\rm s}$, $\rho_{\rm v} \ll \rho_{\rm l}$ and making the ideal gas approximation $p = k_{\rm B}T\rho$, we obtain

$$-(s_{\rm s} - s_{\rm v})|_{\rm sv} = \frac{k_{\rm B}T}{p_{\rm sv}}\frac{dp}{dT}\bigg|_{\rm sv}, \tag{19}$$

and a similar expression for $(s_{\rm l} - s_{\rm v})|_{\rm lv}$. Substituting these into Supplementary Eq. (18), we obtain the sought expression for $d(p_{\rm l} - p_{\rm v})|_{\rm sv}$ explicitly in terms of vapor pressures along sublimation and condensation lines as

$$d(p_{\rm l} - p_{\rm v})|_{\rm sv} = \rho_{\rm l}d\left(k_{\rm B}T\ln\frac{p_{\rm sv}}{p_{\rm lv}}\right). \tag{20}$$

Integrating this equation from the triple point to a desired arbitrary temperature, we obtain

$$\Delta p_{\rm lv}(T)|_{\rm sv} = \rho_{\rm l}k_{\rm B}T\ln\frac{p_{\rm sv}}{p_{\rm lv}}. \tag{21}$$

This is the same result obtained in [16] by alternative means. We use explicit expressions obtained for the vapor pressures of the TIP4P/Ice model to calculate the required pressure difference for use in Supplementary Eq. (4).

SUPPLEMENTARY NOTE 2: ANALYTICAL FORMULA FOR THE SURFACE VAN DER WAALS FORCES

Elbaum and Schick calculated the van der Waals force contributions to the interface potential using Lifshitz theory [15]. The results are obtained only in numerical form from quadrature, which is not convenient for numerical purposes. Here we derive an accurate analytical approximation, along the lines of Ref. [17].

Quite generally, the van der Waals forces between two media, 1 and 2, across a media m enclosed between infinite slabs of media 1 and 2, give rise to an interface potential of the form

$$g_{\rm vdw}(h) = -\frac{A(h)}{12\pi h^2}, \tag{22}$$

where $A(h)$ is the Hamaker function. In a well known approximation to Lifshitz theory, this is given as

$$A(h) = \frac{3}{2} k_{\rm B} T \sum_{n=0}^{\infty\prime} R(\omega_n)[1 + r_n] e^{-r_n}, \tag{23}$$

where the prime indicates that the first term is weighted by a factor of 1/2, $r_n = 2\epsilon_m^{1/2}\omega_n h/c$, $\omega_n = \omega_T n$, $\omega_T = 2\pi k_B T/\hbar$, and ϵ_m is the dielectric constant of the layer of thickness h. The function $R(\omega_n)$ is a complicated expression that depends on the frequency dependent dielectric constants of the material and the film thickness h [17]. For practical purposes, it can be approximated via the simpler expression

$$R(\omega_n) = \left(\frac{\epsilon_1 - \epsilon_m}{\epsilon_1 + \epsilon_m}\right)\left(\frac{\epsilon_2 - \epsilon_m}{\epsilon_2 + \epsilon_m}\right), \tag{24}$$

where ϵ_1 and ϵ_2 are the frequency dependent dielectric constants of the media enclosing the layer of thickness h. At this stage it is convenient to single out the $n = 0$ term in Supplementary Eq. (23), and to further approximate the remaining sum into an integral. Then

$$g_{\rm vdw}(h) = -\frac{A_{\omega=0}}{12\pi h^2} - \frac{A_{\omega>0}(h)}{12\pi h^2}, \tag{25}$$

where

$$A_{\omega=0} = \frac{3}{4}\left(\frac{\epsilon_1 - \epsilon_m}{\epsilon_1 + \epsilon_m}\right)\left(\frac{\epsilon_2 - \epsilon_m}{\epsilon_2 + \epsilon_m}\right) k_{\rm B} T, \tag{26}$$

and

$$A_{\omega>0}(h) = \frac{3\hbar c}{8\pi\epsilon_m^{1/2}} \int_{\nu_T}^{\infty} R(\nu)[1 + \nu h] e^{-\nu h} d\nu, \tag{27}$$

where the sum over angular frequencies has been transformed into an integral over wavenumbers $\nu = 2\epsilon_m^{1/2}\omega/c$ and $\nu_T = 2\epsilon_m^{1/2}\omega_T/c$.

Elbaum and Schick parametrized the dielectric properties of water and ice, and argued that the term $(\epsilon_i - \epsilon_w)$ of the function $R(\nu)$ changes sign at ultra-violet frequencies, such that $R(\nu) < 0$ in the infra-red, but $R > 0$ at the extreme ultra-violet and beyond. In view of this, we split the integral of Supplementary Eq. (27) and write:

$$A_{\omega>0}(h) = \frac{3\hbar c}{8\pi\epsilon_m^{1/2}} \int_{\nu_T}^{\nu_{\rm UV}} R(\nu)[1 + \nu h] e^{-\nu h} d\nu + \frac{3\hbar c}{8\pi\epsilon_m^{1/2}} \int_{\nu_{\rm UV}}^{\infty} R(\nu)[1 + \nu h] e^{-\nu h} d\nu, \tag{28}$$

where $\nu_{\rm UV}$ is the frequency at which $R(\nu)$ is maximum. The first integral can now be evaluated using the first mean value theorem, and the second using the second mean value theorem, yielding

$$\begin{aligned} A_{\omega>0}(h) \;=\; & \frac{3\hbar c}{8\pi\epsilon_m^{1/2} h} R(\nu_{\rm IR})[(2 + \nu_T h)e^{-\nu_T h} - (2 + \nu_{\rm UV} h)e^{-\nu_{\rm UV} h}] \\ & + \frac{3\hbar c}{8\pi\epsilon_m^{1/2} h} R(\nu_{\rm UV})[(2 + \nu_{\rm UV} h)e^{-\nu_{\rm UV} h} - (2 + \nu_\infty h)e^{-\nu_\infty h}]. \end{aligned} \tag{29}$$

This is an exact quadrature for suitably chosen frequencies $\nu_{\rm IR}$ and ν_∞, satisfying $\nu_T < \nu_{\rm IR} < \nu_{\rm UV}$, and $\nu_{\rm UV} < \nu_\infty < \infty$. Collecting terms, the above expression simplifies to

$$A_{\omega>0}(h) = \frac{3\hbar c}{8\pi\epsilon_m^{1/2} h} R(\nu_{\rm IR}) \left[(2 + \nu_T h)e^{-\nu_T h} + (f - 1)(2 + \nu_{\rm UV} h)e^{-\nu_{\rm UV} h} - f(2 + \nu_\infty h)e^{-\nu_\infty h}\right], \tag{30}$$

where $f = R(\nu_{\rm UV})/R(\nu_{\rm IR})$. Supplementary Eq. (30) provides a simple analytic expression which properly captures the crossover from retarded to non retarded interactions, as well as the suppression of retarded interactions at large distances and the temperature dependence of the van der Waals forces.

Assuming that the relevant wave-numbers are well separated, such that $\nu_T \ll \nu_{\rm UV} \ll \nu_\infty$, we find the following four distinct regimes as h increases:

- The subnanometer range, $\nu_\infty h \ll 1$, describes either the $h \to 0$ or $T \to 0$ behavior of $A_{\omega>0}$. Expanding all the exponentials in Supplementary Eq. (30), one finds that the terms of order h^0 inside the square brackets cancel exactly. Retaining then the leading order terms in h, one finds

$$A_{\omega>0}(h) = \frac{3\hbar\omega_\infty}{4\pi} R(\nu_{\rm UV}). \tag{31}$$

  In this regime $A_{\omega>0}$ recovers the standard low temperature asymptotic limit that is well known in the literature. In particularly, $A_{\omega>0}$ is independent of h and one can talk appropriately of a Hamaker constant.

- For $\nu_{\rm UV} h \ll 1 \ll \nu_\infty h$, the last term in Supplementary Eq. (30) is exponentially suppressed, and $A_{\omega>0}$ develops an explicit h dependence

$$A_{\omega>0}(h) = \frac{3\hbar c}{4\pi\epsilon_m^{1/2} h} R(\nu_{\rm UV}). \tag{32}$$

  Using this expression in Supplementary Eq. (22), we recover the standard result for retarded van der Waals interactions. In this range, the free energy has naturally shifted from an h^{-2} to an h^{-3} dependence, while the sign of the interactions remains dominated by the UV dielectric response.

- For $\nu_T h \ll 1 \ll \nu_{\rm UV} h$, the last two terms of Supplementary Eq. (30) are suppressed, and the retarded interactions cross over from an ultraviolet dominated regime, to an infrared dominated regime

$$A_{\omega>0}(h) = \frac{3\hbar c}{4\pi\epsilon_m^{1/2} h} R(\nu_{\rm IR}), \tag{33}$$

  since $R(\nu_{\rm IR})$ and $R(\nu_{\rm UV})$ have opposite signs, the Hamaker function changes sign from positive to negative as the film thickness becomes larger than the cross-over wave-length $\nu_{\rm UV}$ lying in the nanometer length scale.

- Finally, for $\nu_T h \gg 1$, only the first term of Supplementary Eq. (30) remains. This results in an exponentially decaying retarded interaction corresponding to the expected suppression of $A_{\nu>0}$ at microwave distances [18, 19], with

$$A_{\omega>0}(h) = 3k_B T R(\nu_{\rm IR}) e^{-\nu_T h}. \tag{34}$$

For practical purposes, we are only interested in modeling van der Waals forces out to distances of the order of decades of nanometers from the surface, so we assume $\nu_T h \ll 1$, and simplify Supplementary Eq. (30) to

$$g_{\rm vdw}(h) = -\frac{B}{h^3}\left[1 - f\exp(-\nu_{\rm UV} h) - (1-f)\exp(-\nu_\infty h)\right], \tag{35}$$

where now B, f, $\nu_{\rm UV}$ and ν_∞ are parameters chosen to best model the results of Elbaum and Schick in the range of 1 to 10 nm. For sufficiently large $f > 1$, this equation gives the expected crossover in the decay form of $g(h)$ from $\sim h^{-2}$ to $\sim h^{-3}$ dominated regimes found for the ice/water/air interface.

Supplementary Fig. 5 shows a comparison of the exact results from Lifshitz theory together with the fit to Supplementary Eq. (35), showing excellent agreement for the set of parameters displayed in Supplementary Table 1. Since we find that $g_{\rm vdw}(h)$ is a factor of 1/100 smaller than $g_{\rm sr}(h)$ in the range $h < 10$ Å, the van der Waals forces therefore only become relevant at large distances, where $g_{\rm sr}(h)$ becomes negligible due to the exponential decay form that it has.

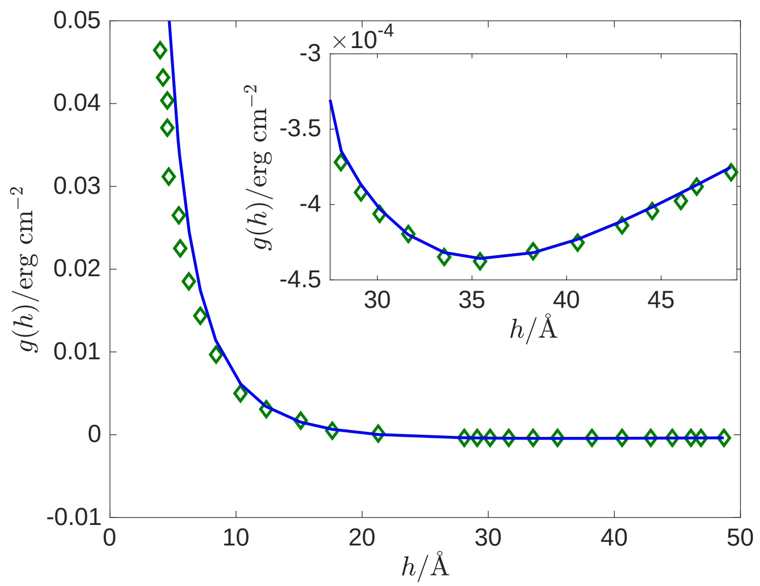

Supplementary Fig. 5: Van der Waals interface potential, as calculated numerically by Elbaum and Schick (symbols), compared with the analytical approximation in Supplementary Eq. (35).

SUPPLEMENTARY NOTE 3: FIT TO THE INTERFACE POTENTIAL

The computer simulation results for the interface potential are fitted to the expression $g(h) = g_{\mathrm{sr}}(h) + g_{\mathrm{vdw}}(h)$, with $g_{\mathrm{sr}}(h)$, the structural short range contribution:

$$g_{\mathrm{sr}}(h) = C_2 \exp(-\kappa_2 h) - C_1 \exp(-\kappa_1 h) \cos(q_0 h + \alpha) \tag{36}$$

We use the coefficients C_i, κ_2, κ_1, q_0 and α as fitting parameters, setting $\kappa_2 = 2\kappa_1$, for simplicity. Since the interface potential obtained from simulation is exact up to an additive constant, we seek parameters by minimizing the least square deviations from the corresponding disjoining pressure $\Pi(h) = -\partial_h g(h)$. We include also a constraint in the minimization to force the minimum of the interface potential to be at $g_{\mathrm{min}} = -5.9 \times 10^{-5}$ J/m^2, consistent with the observed contact angle of a droplet on an α film. The parameter values obtained from this fitting may be found in the Supplementary Table 1. The value found for q_0 is consistent with a strong renormalization away from the value one would expect from mean field theory [20–22]. In our fits we find that the target depth of the primary minimum of the interface potential could not be reached. Since the separation between the kinetic phase lines is dictated by the depth and free energy difference of the minima, the phase lines in Fig. 4 (main text) appear very close to each other. To illustrate the role of the well depth in separating the phase lines, Supplementary Fig. 1 uses an interface potential blown up by a factor of 30. The resulting phase lines very much resemble the kinetic phase diagram observed experimentally [23]. A recent study indicates that the van der Waals forces estimated by Elbaum and Schick used here suffer from insufficient optical data and predict interactions that are one order of magnitude too weak [24, 25]. This explains why the energy minima of our model interface potential are so shallow and produce such a small separation between the kinetic phase lines.

SUPPLEMENTARY NOTE 4: STOCHASTIC DYNAMICS OF THE SINE GORDON + CAPILLARY WAVE MODEL

Consider a microscopic realization of the premelting film at a given instant, obtained with atomistic detail e.g. in a Molecular Dynamics simulation. The detailed state of this system, as given by the atomic positions, may be conveniently described by two interface profiles $\hat{L}_{\rm sl}(x,t)$ and $\hat{L}_{\rm lv}(x,t)$ of the solid/liquid and liquid/vapor interfaces by using a suitable coarse-graining on the scale of a few molecular diameters [16, 26–28]. Thus, these profiles are still microscopic scale quantities.

In the spirit of the Langevin equation, we expect that the time evolution of the interface profiles will be dictated by two different processes. First, a deterministic evolution that is driven by a coarse-grained Hamiltonian, $\mathcal{H}[\hat{L}_{\rm sl},\hat{L}_{\rm lv}]$. Second, a Gaussian random evolution that describes the thermal fluctuations of the coarse-grained degrees of freedom. Such a dynamical equation for a thin liquid film on an inert substrate was derived in [29]. Here, using heuristic arguments we generalise this to the present case of a water film on ice with evaporation/condensation and freezing/melting.

The time evolution at the solid/liquid surface is the result of freezing and melting events, which can be described by a non-conserved dynamics of $\hat{L}_{\rm sl}(x,t)$ as:

$$\frac{\partial \hat{L}_{\rm sl}}{\partial t} = -k_{\rm sl}\frac{\delta \mathcal{H}}{\delta \hat{L}_{\rm sl}} + R_{\rm sl}(x,t) \tag{37}$$

where $R_{\rm sl}(x,t)$ is a white noise field that accounts for microscopic detailed balance at equilibrium.

Similarly, the time evolution at the liquid/vapor surface is the result of condensation and evaporation of the liquid film, which is conventionally described in terms of a non-conserved dynamics. However, since the premelting film is fluid, we must also account for the spreading dynamics of the film, which we can describe using the thin film approximation. Taking together the two processes, this leads to a generalization of the stochastic thin film equation in the presence of evaporation/condensation:

$$\frac{\partial \hat{L}_{\rm lv}}{\partial t} = \left[\nabla\cdot\frac{\hat{h}^3}{3\eta}\nabla - k_{\rm lv}\right]\frac{\delta \mathcal{H}}{\delta \hat{L}_{\rm lv}} + R_{\rm lv}(x,t) \tag{38}$$

where we have introduced $\hat{h} = \hat{L}_{\rm lv} - \hat{L}_{\rm sl}$ for short, while $R_{\rm lv}(x,t)$ is a noise field that accounts for random stress fluctuations within the premelting film, together with random evaporation and condensation events at the liquid/vapor surface [29]. Notice the model assumes the lubrication approximation for the advective dynamics of the thin liquid film, which is accurate provided the characteristic wavelength of the lateral height variations is larger than the thickness of the liquid layer. This condition is obeyed for the very small contact angle droplets which is certainly the case for the system of interest here.

Of course, the evolution of the two surfaces is not independent, and leads to a pair of coupled stochastic differential equations for the dynamics of the premelting film:

$$\frac{\partial \hat{L}_{\rm sl}}{\partial t} = -k_{\rm sl}\frac{\delta \mathcal{H}}{\delta \hat{L}_{\rm sl}} + \sqrt{2k_{\rm B}Tk_{\rm sl}}\,\xi_{\rm sl}(x,t), \tag{39}$$

$$\frac{\partial \hat{L}_{\rm lv}}{\partial t} = \nabla\cdot\left[\frac{\hat{h}^3}{3\eta}\nabla\frac{\delta \mathcal{H}}{\delta \hat{L}_{\rm lv}} + \sqrt{\frac{2k_{\rm B}T\hat{h}^3}{\eta}}\,\xi_{\rm tf}(x,t)\right] - k_{\rm lv}\frac{\delta \mathcal{H}}{\delta \hat{L}_{\rm lv}} + \sqrt{2k_{\rm B}Tk_{\rm lv}}\,\xi_{\rm lv}(x,t) - \frac{\Delta\rho}{\rho_{\rm l}}\frac{\partial \hat{L}_{\rm sl}}{\partial t}. \tag{40}$$

Notice that, on account of the premelting film's incompressibility, changes in $\hat{L}_{\rm sl}$ are conveyed into $\hat{L}_{\rm lv}$, so that the full dynamics of $\hat{L}_{\rm lv}$ is dictated both by condensation/evaporation and freezing/melting rates. The stochastic nature of the growth process is described by spatially and temporal uncorrelated white noise fields, $\xi_{\rm sl}$, $\xi_{\rm lv}$ and $\xi_{\rm tf}$ that describe coarse-grained thermal fluctuations at the solid/liquid surface, the liquid/vapor surface and the premelting film, respectively. Finally, the amplitude of the random noise is chosen such that linearized forms of Eq. (37) and Eq. (38) at equilibrium satisfy the fluctuation-dissipation theorem exactly (i.e. obey detailed balance).

In order to be more specific, we now consider an explicit form for the Hamiltonian, based on the sine Gordon model for the description of the solid/liquid surface, and the capillary wave Hamiltonian for the description of the liquid/vapor surface [26–28]:

$$\mathcal{H}[\hat{L}_{\rm sl},\hat{L}_{\rm lv}] = \int\left[\frac{\gamma_{\rm sl}}{2}(\nabla\hat{L}_{\rm sl})^2 + \frac{\gamma_{\rm lv}}{2}(\nabla\hat{L}_{\rm lv})^2 - u\cos(q_z\hat{L}_{\rm sl}) + g(\hat{L}_{\rm lv}-\hat{L}_{\rm sl}) - \Delta p_{\rm sl}\hat{L}_{\rm sl} - \Delta p_{\rm lv}\hat{L}_{\rm lv}\right]{\rm d}\mathbf{x}, \tag{41}$$

where $\gamma_{\rm sl}$, the solid/liquid stiffness coefficient and $\gamma_{\rm lv}$, the water/vapor surface tension penalize the increase of surface area; the cosine term favors solid/liquid film heights that are congruent with the crystal lattice spacing as dictated by the wave-vector q_z; u dictates the energy cost for excursions away from the preferred spacing; $g(\hat{L}_{\rm lv} - \hat{L}_{\rm sl})$ is the interface potential, which sets the equilibrium film height at coexistence; and finally, $\Delta p_{\rm sl} = p_{\rm s} - p_{\rm l}$ and $\Delta p_{\rm lv} = p_{\rm l} - p_{\rm v}$, with p_α the bulk pressure of phase α are fields which account for the free energy cost of forming a liquid film at the expense of solid and vapor phases, respectively. Notice however that the coefficients of this Hamiltonian are 'bare' or mean field parameters obtained from a microscopic theory averaged on the scale of the bulk correlation length.

Using the above Hamiltonian together with Eqs. (39) and (40), we obtain the following explicit equation for the stochastic evolution of the coupled sine Gordon + Capillary Wave model:

$$\frac{\partial \hat{L}_{\rm sl}}{\partial t} = -k_{\rm sl}[\gamma_{\rm sl}\nabla^2 \hat{L}_{\rm sl} + w\sin(q_z \hat{L}_{\rm sl}) - \phi_{\rm sl}] + \sqrt{2k_BTk_{\rm sl}}\xi_{\rm sl}(x,t), \tag{42}$$

$$\frac{\partial \hat{L}_{\rm lv}}{\partial t} = (\nabla\cdot\frac{\hat{h}^3}{3\eta}\nabla + k_{\rm lv})[\gamma_{\rm lv}\nabla^2\hat{L}_{\rm lv} + \phi_{\rm lv}] + \nabla\cdot\sqrt{\frac{2k_BT\hat{h}^3}{3\eta}}\xi_{\rm tf}(x,t) + \sqrt{2k_BTk_{\rm lv}}\xi_{\rm lv}(x,t) - \frac{\Delta\rho}{\rho_{\rm l}}\frac{\partial \hat{L}_{\rm sl}}{\partial t}, \tag{43}$$

where $w = q_z u$, $\phi_{\rm sl} = \Delta p_{\rm sl} - \Pi$ and $\phi_{\rm lv} = \Delta p_{\rm lv} + \Pi$, with Π the disjoining pressure, defined as $\Pi(h) = -dg(h)/dh$.

This result may be considered as a generalized stochastic thin film equation [29–31] that accounts also for variations of the underlying substrate by means of the sine Gordon model [32–34], and condensation/evaporation by means of a growth equation for rough surfaces [35]. For inert substrates and non volatile liquids ($k_{\rm sl} = k_{\rm lv} = 0$), it reduces exactly to the stochastic thin film equation [29–31]. For the buried solid substrate below an infinitely thick liquid film in equilibrium ($\hat{L}_{\rm lv} = D$, with the constant $D \to \infty$), it reduces exactly to the stochastic sine Gordon equation [32–34, 36] and for an infinitely viscous premelting film ($\eta = \infty$) under a flat inert substrate ($k_{\rm sl} = 0$) it recovers the stochastic growth equation of rough interfaces [35]. In each of these limiting cases, the amplitude of the noise is set such that the fluctuation-dissipation theorem is obeyed exactly.

Obviously, the model does not incorporate any effects related to thermal gradients. However, it is believed that for films less than approximately 100 nm, disjoining pressure effects largely dominate over thermo-capillary forces [37]. Furthermore, the experiments we describe appear to fulfil local thermal equilibrium, since growth and evaporation events appear to be reversible and reproducible [1].

Within this premise, the above result incorporates many details of the physics. For equilibrium systems, with no forcing, the stochastic dissipative equations serves as a starting point for renormalization of the bare Hamiltonian [34, 38, 39]. For systems out of equilibrium, it describes correctly purely dissipative processes that occur deterministically when very large free energy gradients are present. Thanks to the stochastic term, it can also describe excursions away from the deterministic path when thermal fluctuations are comparable to the energy gradients, and is also able to describe uphill activated processes against the free energy gradients. In particular, the stochastic thin film equation is able to predict the nucleation of metastable thin films [29], while the stochastic sine Gordon model can describe terrace nucleation and activated crystal growth of faceted surfaces [33, 36], as well as spiral growth [40].

Unfortunately, this detailed stochastic description can be in practice rather cumbersome, as very lengthy simulations are required to observe activated processes, while the stochastic nature of the dynamics implies the need to collect averages over a large ensemble of trajectories.

For this reason, it is convenient to perform an average of Eqs. (42) and (43) over the set of all random realizations of the noise subject to a given initial condition, in a manner similar to that performed in Dynamical Density Functional Theory [41, 42]. The reward for this additional averaging is that the resulting evolution equation becomes deterministic, and we can then avoid studying a large number of trajectories and implementing the cumbersome details of stochastic differential equations.

To see this, assume that $L_{\rm sl}(x,t)$ and $L_{\rm lv}(x,t)$ are the noise averaged film profiles. Then, a given realization of the stochastic evolution as described by Eqs. (42) and (43) may be expressed in terms of deviations away from the averaged profile as $\hat{L}_{\rm sl}(x,t) = L_{\rm sl}(x,t) + \delta\hat{L}_{\rm sl}(x,t)$, and likewise for $\hat{L}_{\rm lv}(x,t)$. Plugging these into Eqs. (42) and (43), and performing an average over all realizations of the noise, linear terms in $\hat{L}_{\rm sl}$ are immediately transformed into terms with exactly the same form in $L_{\rm sl}$, by definition, while the random noise terms vanish. Therefore:

$$\frac{\partial L_{\rm sl}}{\partial t} = -k_{\rm sl}[\gamma_{\rm sl}\nabla^2 L_{\rm sl} + w\langle\sin(q_z\hat{L}_{\rm sl})\rangle_\xi - \langle\phi_{\rm sl}\rangle_\xi], \tag{44}$$

$$\frac{\partial L_{\rm lv}}{\partial t} = \langle(\nabla\cdot\frac{\hat{h}^3}{3\eta}\nabla + k_{\rm lv})[\gamma_{\rm lv}\nabla^2\hat{L}_{\rm lv} + \phi_{\rm lv}]\rangle_\xi - \frac{\Delta\rho}{\rho_{\rm l}}\frac{\partial L_{\rm sl}}{\partial t}, \tag{45}$$

where $\langle\rangle_\xi$ stands here for the average over the random trajectories. In the spirit of the Smoluchowski equation, the ensemble average over trajectories can be replaced by an ensemble average over the film fluctuations, $\delta\hat{L}_{\rm sl}(x,t)$ and $\delta\hat{L}_{\rm lv}(x,t)$. Then, assuming local thermal equilibrium during the time evolution of the averaged profile, the random film height deviations $\delta\hat{L}_{\rm sl}(x,t)$ and $\delta\hat{L}_{\rm lv}(x,t)$ are Gaussian distributed, with a variance that is given by the equilibrium thermal fluctuations. Accordingly, the ensemble over trajectories may be replaced by a canonical ensemble over fluctuations $\langle\rangle_\xi \to \langle\rangle_T$.

In this approximation, we can readily interpret the first of the coupled equations. The exact Gaussian average of $\sin(q_z\hat{L}_{\rm sl})$ readily yields:

$$\langle\sin(q_z\hat{L}_{\rm sl})\rangle_T = e^{-\frac{1}{2}q_z^2\langle\delta\hat{L}_{\rm lv}^2\rangle_T}\sin(q_z L_{\rm sl}). \tag{46}$$

Accordingly, the sinusoidal term $w\sin q_z\hat{L}_{\rm sl}$ of the original Hamiltonian, that is a function of the microscopic film profile $\hat{L}_{\rm sl}$, is transformed exactly into a sinusoidal term that is a function of the averaged film profile $L_{\rm sl}$, albeit with a renormalized amplitude $we^{-\frac{1}{2}q_z^2\langle\delta\hat{L}_{\rm lv}^2\rangle}$ [34, 43, 44]. At a higher level of approximation, also the surface tension $\gamma_{\rm sl}$ is renormalized [32, 38], but this difficulty need not concern us here since this level of renormalization predicts the location of the roughening transition exactly.

On the other hand, the Gaussian average of $\phi_{\rm sl}(\hat{h}) = \Delta p_{\rm sl} - \Pi(\hat{h})$ yields:

$$\langle\phi_{\rm sl}(\hat{h})\rangle_T = \Delta p_{\rm sl} - \langle\Pi(\hat{h})\rangle_T \tag{47}$$

where $\langle\Pi(\hat{h})\rangle$ is the Gaussian renormalized disjoining pressure. In our model, the disjoining pressure consists of short ranged (exponentially decaying) terms, and an algebraically decaying term. The latter does not renormalize, because of the long range nature, and need not concern us any longer [45] while the former can be worked out exactly under Gaussian renormalization [20, 22]. The result is again formally equal to the bare disjoining pressure of the Hamiltonian, albeit with renormalized coefficients, as discussed in the main text.

It follows that the dynamics of $L_{\rm sl}$ may be cast as:

$$\frac{\partial L_{\rm sl}}{\partial t} = -k_{\rm sl}[\gamma_{\rm sl}\nabla^2 L_{\rm sl} + w_{\rm R}\sin(q_z L_{\rm sl}) - \Delta p_{\rm sl} + \Pi_{\rm R}(h)] \tag{48}$$

where the subscript R stands for Gaussian renormalized quantities. This equation is formally identical to Eq. (42), albeit with the microscopic film heights replaced by average film heights, and the bare parameters of the Hamiltonian replaced by renormalized coefficients.

A similar result for the time evolution of $L_{\rm lv}$ cannot be readily obtained, because the mobility coefficient in the lubrication approximation depends on the film thickness. Hence, the Gaussian average of the Hamiltonian couples with the $\hat{h}^3$ term in the mobility coefficient. However, in our system the fluctuations of $\hat{L}_{\rm sl}$ are smaller than one lattice spacing because the surface is smooth, while the fluctuations of $\hat{L}_{\rm lv}$ are limited by the long range van der Waals tail and increase logarithmically with the film height. Accordingly, we expect that the fluctuations of $\hat{h}$ away from the mean value h will be small. This allows us to expand the mobility in powers of $\delta\hat{h}$ and retain only the leading order term. The time evolution of $L_{\rm lv}$ is then given as:

$$\frac{\partial L_{\rm lv}}{\partial t} = (\nabla\cdot\frac{h^3}{3\eta}\nabla + k_{\rm lv})[\gamma_{\rm lv}\nabla^2 L_{\rm lv} + \Delta p_{\rm lv} + \Pi_R(h)] \tag{49}$$

where again the subscript R stands for Gaussian renormalized quantities.

Eq. (48) and Eq. (49) provide a system of deterministic coupled differential equations for the average dynamics of the coupled stochastic sine Gordon and stochastic thin film equations:

$$\frac{\partial L_{\rm sl}}{\partial t} = -k_{\rm sl}[\gamma_{\rm sl}\nabla^2 L_{\rm sl} + w\sin(q_z L_{\rm sl}) - \phi_{\rm sl}], \tag{50}$$

$$\frac{\partial L_{\rm lv}}{\partial t} = (\nabla\cdot\frac{h^3}{3\eta}\nabla + k_{\rm lv})[\gamma_{\rm lv}\nabla^2 L_{\rm lv} + \phi_{\rm lv}] - \frac{\Delta\rho}{\rho_{\rm l}}\frac{\partial L_{\rm sl}}{\partial t}. \tag{51}$$

From Eqs. (50) and (51) we can readily see that the trajectory averaged film profiles $L_{\rm sl}$ and $L_{\rm lv}$ follow a time evolution that is exactly the same as that for the microscopic profiles $\hat{L}_{\rm sl}$ and $\hat{L}_{\rm lv}$ given in Eqs. (42) and (43), albeit with renormalized parameters of the Hamiltonian.

In view of this, we can write the coupled time evolution of L_{sl} and L_{lv} as in Eq. (3) of the main manuscript, with the understanding that the free energy, Ω is a Gaussian renormalized Hamiltonian $\Omega = \langle \mathcal{H} \rangle_{\xi} \approx \langle \mathcal{H} \rangle_{T}$, which adopts the same form as the mean field Hamiltonian, Eq. (41), albeit with renormalized coefficients.

This interpretation is very much analogous to the similar results found in Dynamic Density Functional Theory [41, 42, 46], which is a theory for the dynamics of the density distribution of Brownian stochastic particles (i.e. colloids). There, the resulting deterministic equation is an evolution equation for the average density profile, obtained by averaging over all realizations of the noise, while the stochastic equation describes the evolution of the microscopic density for one particular realization of the noise. The input to the deterministic equation is the free energy functional known from equilibrium Density Functional Theory [21]. In this picture, the deterministic evolution of the microscopic density can also be interpreted to describe the most likely path of the stochastic process when the fluctuations are small [46, 47]. In this case, the microscopic Hamiltonian does not deviate significantly from the renormalized free energy, and then the average evolution is essentially the same as that of the most likely path, as expected in mean field.

SUPPLEMENTARY NOTE 5: MEAN FIELD DYNAMICS AND KINETIC PHASE DIAGRAM

The dynamics of the premelting film, i.e. of the solid/liquid and liquid/vapor interfaces $L_{\rm sl}$ and $L_{\rm lv}$ respectively, is governed by the free energy in Eq. (3) of the main text, together with the gradient dynamics equations in Eq. (4) of the main text.

The dynamics exhibited by this pair of coupled partial differential equations is very rich, and the full gamut can only be found by solving numerically. However, analytic results can be obtained for the long-time average behavior, i.e. for the growth speeds. For $\phi_{\rm sl}^2 < w^2$, ice growth (corresponding to $L_{\rm sl}$ increasing) cannot occur, because the thermodynamic force $\phi_{\rm sl}$ is not sufficient to overcome the sinusoidal pinning potential. Therefore, growth proceeds by the horizontal spread of terraces with velocity $\frac{\pi}{4} k_{\rm sl} (\frac{\gamma_{\rm sl}}{u})^{1/2} \phi_{\rm sl}$ [34, 38]. For $\phi_{\rm sl}^2 > w^2$, the driving potential $\phi_{\rm sl}$ overcomes the sinusoidal potential, and uniform growth can occur. However, if $\phi_{\rm sl}$ is only marginally larger than w, the process occurs in a stepwise fashion, with a long interval in which there is almost no growth, followed by fast growth over a short time period, as observed in computer simulations [48]. This leads to a height increment of $\approx 2\pi/q_z$, i.e. of one ice lattice spacing. The process repeats recursively with a period $\tau = 2\pi/q_z \sqrt{\phi_{\rm sl}^2 - w^2}$, so that the average growth rate is $k_{\rm sl}\sqrt{\phi_{\rm sl}^2 - w^2}$. For large $\phi_{\rm sl}$, this provides the usual 'linear growth' mode of rough interfaces, but in the limit $\phi_{\rm sl} \approx w$, the linear growth mode can be much slower than the horizontal translation of terraces.

For flat films, the average growth rate over time scales much larger than τ is then given by

$$\langle \frac{\partial L_{\rm sl}}{\partial t} \rangle = \pm k_{\rm sl} \sqrt{\phi_{\rm sl}^2 - w^2} \tag{52}$$

$$\langle \frac{\partial L_{\rm lv}}{\partial t} \rangle = k_{\rm lv}\phi_{\rm lv} - \frac{\Delta\rho}{\rho_{\rm l}} \langle \frac{\partial L_{\rm sl}}{\partial t} \rangle \tag{53}$$

where the plus sign stands for freezing, and the minus sign for sublimation. Subtracting one from the other, we obtain the average speed of the liquid film thickness growth

$$\langle \frac{\partial h}{\partial t} \rangle = k_{\rm lv}\phi_{\rm lv} \mp \frac{\rho_{\rm s}}{\rho_{\rm l}} k_{\rm sl} \sqrt{\phi_{\rm sl}{}^2 - w^2}. \tag{54}$$

This result becomes particularly simple for the case when $w = 0$, as discussed in the text. In the general case where $w \neq 0$ and $p > p_{\rm sv}$ so that the height of the ice grows, the condition that the liquid thickness is stationary $\langle \partial_t h \rangle = 0$ is achieved for $\phi_{\rm lv} \geq 0$, $\phi_{\rm sl} \geq 0$, and $\phi_{\rm sl}^2 - w^2 \geq 0$. In the marginal case where $\phi_{\rm sl} = w$, then we need $\phi_{\rm lv} = 0$. Solving these two conditions simultaneously corresponds to $\Delta p_{\rm sl} + \Delta p_{\rm lv} = \pm w$. Using the approximate but nonetheless accurate thermodynamic relations for the pressure differences given below in Eqs. (68) and (69), these condition may be solved as a function of T, yielding the following equation for the boundary

$$p_{\rm ns}(T) = p_{\rm sv}(T) e^{\pm \frac{w}{\rho_{\rm s} k_{\rm B} T}}. \tag{55}$$

States between the sublimation line $p_{\rm sv}(T)$ and the boundary line $p_{\rm ns}(T)$ neither grow nor sublimate because the surface $L_{\rm sl}$ can not grow in the absence of thermal activation.

For the more general case when $\phi_{\rm sl}^2 - w^2 \geq 0$, the stationarity condition is achieved as a solution of the equation

$$k_{\rm lv}\phi_{\rm lv} \mp \frac{\rho_{\rm s}}{\rho_{\rm l}} k_{\rm sl} \sqrt{\phi_{\rm sl}^2 - w^2} = 0. \tag{56}$$

It corresponds to the condition that the liquid/vapor and solid/liquid surfaces grow at the same rate. Only one solution exists, given that the surface growth rates are monotonic. However, in order to solve explicitly we need to square each term. The resulting equation then has two solutions, each of the same magnitude but with opposite sign. Of course, one is unphysical. Therefore, squaring in Eq. (56) we obtain

$$\rho_{\rm s}^2 k_{\rm sl}^2 \phi_{\rm sl}^2 - \rho_{\rm l}^2 k_{\rm lv}^2 \phi_{\rm lv}^2 = \rho_{\rm s}^2 k_{\rm sl}^2 w^2, \tag{57}$$

under the condition that $p_{\rm v} > p_{\rm sl}(T)$. This provides a quadratic equation for Π as a function of $p_{\rm v}$ and T, so one obtains

$$\Pi = -\Delta p_{\rm k}, \tag{58}$$

with

$$\Delta p_{\rm k} = -\frac{f_{\rm s}^2 \Delta p_{\rm sl} + f_{\rm l}^2 \Delta p_{\rm lv} \pm f_{\rm s} \left[f_{\rm l}^2 (\Delta p_{\rm sl} + \Delta p_{\rm lv})^2 + (f_{\rm s}^2 - f_{\rm l}^2) w^2 \right]^{1/2}}{(f_{\rm s}^2 - f_{\rm l}^2)}, \tag{59}$$

where $f_\mathrm{s} = \rho_\mathrm{s} k_\mathrm{sl}$ and $f_\mathrm{l} = \rho_\mathrm{l} k_\mathrm{lv}$. Thus, the solution may formally be written in exactly the same form as the equilibrium condition for the adsorption on an inert substrate, with the Laplace pressure difference $\Delta p = p_\mathrm{l} - p_\mathrm{v}$ replaced by a kinetic pressure difference Δp_k which depends on the growth mechanism and rate constants. Likewise, an effective potential exists whose extrema are stationary states of the underlying dynamics.

Alternatively, Eq. (57) may be solved for p_v as a function of Π and T, with the result:

$$\rho_\mathrm{l} k_\mathrm{B} T \ln \frac{p}{p_\mathrm{lv}} + \Pi = -\frac{\Delta\rho C \pm \left[\kappa^2 \rho_\mathrm{l}^2 C^2 + \rho_\mathrm{l}^2 w^2 (\Delta\rho^2 - \kappa^2 \rho_\mathrm{l}^2)\right]^{1/2}}{\Delta\rho^2 - \kappa^2 \rho_\mathrm{l}^2}, \tag{60}$$

where $\kappa = \rho_\mathrm{l} k_\mathrm{lv} / \rho_\mathrm{s} k_\mathrm{sl}$ and $C = \rho_\mathrm{s} \rho_\mathrm{l} k_\mathrm{B} T \ln \frac{p_\mathrm{lv}}{p_\mathrm{sv}} - \rho_\mathrm{s} \Pi$. In this case, the result corresponding to $w = 0$ and $\Pi = 0$ (for a rough ice surface) is obtained for the '+' root. Three kinetic transition lines in the phase diagram can be identified from the numerical solution of these equations as discussed in the main text. Particularly, the kinetic coexistence line between α and β states obeys a double tangent construction of wetting phase diagrams, albeit with the kinetic overpressure replacing the equilibrium value:

$$\omega_\mathrm{k}(h_1) = \omega_\mathrm{k}(h_2) \tag{61}$$

$$\Pi(h_1) = -\Delta p_\mathrm{k} \tag{62}$$

$$\Pi(h_2) = -\Delta p_\mathrm{k} \tag{63}$$

The first condition imposes equal effective free energy for both films, and the other two impose that both states obey the quasi-stationary condition at equal kinetic overpressure $-\Delta p_\mathrm{k}$. Alternatively, these equations may be written more concisely as:

$$g(h_1) + \Pi(h_1) h_1 = g(h_2) + \Pi(h_2) h_2 \tag{64}$$

$$\Pi(h_1) = \Pi(h_2) \tag{65}$$

Once the value of Π that satisfies the condition is known, the pressure p_v at which the condition is met can be obtained by solving Eq. (57) for $p_\mathrm{v}(T)$ using the appropriate value of Π in Eq. (60).

From these observations we are able to construct the highly detailed kinetic phase-diagram shown in Fig. 4 of the main text, which is an essential tool for understanding at different state points the numerical results obtained from the coupled gradient dynamics partial differential equations in Eq. (4) of the main text.

SUPPLEMENTARY NOTE 6: THERMODYNAMIC FUNCTIONS AND THE EQUILIBRIUM PHASE DIAGRAM

The pressure differences between solid/liquid and liquid/vapor phases are the thermodynamic driving forces that lead to the growth of the ice and the liquid from the vapor. To determine these differences requires knowledge of the equilibrium phase diagram, i.e. to know the pressure as a function of temperature along the condensation and sublimation lines, $p_{\mathrm{sl}}(T)$ and $p_{\mathrm{sv}}(T)$, respectively. We obtain these by assuming they follow from the Clausius-Clapeyron equation. This approximation is excellent for the sublimation line [49], and remains good for the vaporization line down to 260 K [4]. They are given by

$$\ln \frac{p_{\mathrm{sv}}}{p_{\mathrm{t}}} = \frac{\Delta H_{\mathrm{sv}}}{R}\left(\frac{1}{T_{\mathrm{t}}} - \frac{1}{T}\right), \tag{66}$$

$$\ln \frac{p_{\mathrm{lv}}}{p_{\mathrm{t}}} = \frac{\Delta H_{\mathrm{lv}}}{R}\left(\frac{1}{T_{\mathrm{t}}} - \frac{1}{T}\right), \tag{67}$$

where T_{t} and p_{t} are the temperature and pressure at the triple point, R is the gas constant, ΔH_{sv} is the molar enthalpy change for sublimation and ΔH_{lv} is the molar enthalpy change for condensation.

Since ice and water can both be treated as effectively being incompressible, the pressure changes which are relevant to this study are very small. Therefore, the pressure differences can accurately be approximated by

$$p_{\mathrm{l}} - p_{\mathrm{v}} = \rho_{\mathrm{l}} RT \ln \frac{p_{\mathrm{v}}}{p_{\mathrm{lv}}(T)}, \tag{68}$$

$$p_{\mathrm{s}} - p_{\mathrm{v}} = \rho_{\mathrm{s}} RT \ln \frac{p_{\mathrm{v}}}{p_{\mathrm{sv}}(T)}. \tag{69}$$

Using Eqs. (66)–(69), we obtain explicit expressions for the liquid-vapor and ice-liquid overpressures as

$$p_{\mathrm{l}} - p_{\mathrm{v}} = \rho_{\mathrm{l}} RT \ln \frac{p_{\mathrm{v}}}{p_{\mathrm{t}}} - \frac{\rho_{\mathrm{l}} \Delta H_{\mathrm{lv}}(T - T_{\mathrm{t}})}{T_{\mathrm{t}}}, \tag{70}$$

$$p_{\mathrm{s}} - p_{\mathrm{l}} = (\rho_{\mathrm{s}} - \rho_{\mathrm{l}}) RT \ln \frac{p_{\mathrm{v}}}{p_{\mathrm{t}}} + \frac{(\rho_{\mathrm{l}} \Delta H_{\mathrm{lv}} - \rho_{\mathrm{s}} \Delta H_{\mathrm{sv}})(T - T_{\mathrm{t}})}{T_{\mathrm{t}}}. \tag{71}$$

Notice that the pressure difference between the solid and liquid phases decreases as the ambient vapor pressure increases. The triple point data required for the implementation of Eqs. (66)–(71) may be found in Supplementary Table 2.

SUPPLEMENTARY NOTE 7: KINETIC COEFFICIENTS FOR THE GROWTH RATE LAWS

Growth of the liquid/vapor surface

For an infinitely thick premelting film with a flat liquid-vapor surface, Eq. (50) for the growth rate of the surface becomes

$$\frac{\partial L_{\rm lv}}{\partial t} = k_{\rm lv}\Delta p_{\rm lv}. \tag{72}$$

Replacing $p_{\rm l} - p_{\rm v} \approx \rho_{\rm l} k_{\rm B} T (p - p_{\rm lv})/p_{\rm lv}$ in the term for condensation/evaporation rate, we find

$$\frac{\partial L_{\rm lv}}{\partial t} \approx k_{\rm lv}\rho_{\rm l} k_{\rm B} T (p - p_{\rm lv})/p_{\rm lv}. \tag{73}$$

This result can be compared to the Knudsen-Hertz law, which reads

$$\frac{\partial L_{\rm lv}}{\partial t} = k_{\rm KH}(p - p_{\rm lv}), \tag{74}$$

where $k_{\rm KH} = \alpha_{\rm lv}/\rho_{\rm l}(2\pi m_{\rm w} k_B T)^{1/2}$, and where $\alpha_{\rm lv}$ is the sticking coefficient, or fraction of vapor molecules that stick to the interface upon collision and $m_{\rm w}$ is the mass of a water molecule. Therefore, we find

$$k_{\rm lv} = \frac{p_{\rm lv}}{\rho_{\rm l} k_{\rm B} T} k_{\rm KH}. \tag{75}$$

We calculate $k_{\rm lv}$ using the thermodynamic data reported in Supplementary Table 2. We also assume $\alpha_{\rm lv} = 1$ for the attachment of pure water vapor onto the ice surface, consistent with all current molecular simulation studies [50–53].

Growth of the solid/liquid surface

For an infinitely thick premelting film with flat solid-liquid interface, Eq. (50) for the growth rate of the surface becomes

$$\frac{\partial L_{\rm sl}}{\partial t} = k_{\rm sl}\Delta p_{\rm sl}. \tag{76}$$

Replacing $p_{\rm s} - p_{\rm v} \approx \rho_{\rm s}\Delta H_{\rm sl}\frac{T - T_{\rm t}}{T_{\rm t}}$ in the term for the freezing/melting rate we find

$$\frac{dL_{\rm sl}}{dt} \approx k_{\rm sl}\rho_{\rm s}\Delta H_{\rm sl}\frac{T_{\rm t} - T}{T_{\rm t}}. \tag{77}$$

This result can be compared to the law of linear growth for a crystal from the melt which holds at large undercooling [5],

$$\frac{dL_{\rm sl}}{dt} = k_{\rm LG}(T_{\rm t} - T). \tag{78}$$

The result for the rate constant suggested by Librecht [54], $k_{\rm LG} = 0.07$ cm/s K, leads to $k_{\rm sl} = 6 \times 10^{-10}$ m/s Pa. However, the slope of the kinetic coexistence line is determined by the ratio $k_{\rm sl}/k_{\rm lv}$, and we find that the slopes observed in experiments can only be reproduced for $k_{\rm sl}/k_{\rm lv} \approx 6.4$, which is about a factor of 10 smaller. It seems likely that the kinetic coefficient for growth from the premelting film could be significantly smaller than that from the melt, since the interface is considerably smoother [26]. Therefore, in our calculations we set $k_{\rm sl} = 6.4 k_{\rm lv}$.

Size of the region where nucleated dynamics occurs

For $h \to \infty$, our model gives an equation for the dynamics of $L_{\rm sl}$ that corresponds to the growth of ice within supercooled water. This is

$$\frac{\partial L_{\rm sl}}{\partial t} = k_{\rm sl}(\gamma_{\rm sl}\nabla^2 L_{\rm sl} - u q_z \sin(q_z L_{\rm sl}) + \Delta p_{\rm sl}), \tag{79}$$

which is a forced overdamped sine-Gordon equation. The growth is nucleated for $uq_z > \Delta p_{\rm sl}$, and otherwise linear in time [36, 55]. Therefore, we can obtain an order of magnitude estimate for the parameter u from the value of the temperature where there is a crossover from nucleated to linear growth of ice in supercooled water. According to Pruppacher [5], this occurs at about $T - T_{\rm t} \approx 2$ K. Using $p_{\rm s} - p_{\rm l} = \rho_{\rm s} \Delta H_{\rm sl} \Delta T / T_{\rm t}$, we find

$$u = \frac{d_{\rm B}}{2\pi} \rho_{\rm s} \Delta H_{\rm sl} \frac{\Delta T^*}{T_{\rm t}}. \tag{80}$$

Using $\Delta T^* = 2$ K as suggested from results in Ref. [56], and $d_{\rm B} = 0.37$ nm, we find $u = 1.3 \times 10^{-4}$ J/m^2. This is about five times larger than the results obtained from computer simulations, which yield $u = 2.8 \times 10^{-5}$ J/m^2 [26–28]. The value we use is given in Supplementary Table 3.

Viscosity

In principle, the lubrication approximation on which our thin film dynamics model is based on uses as input the bulk liquid viscosity. Some studies suggest there is a large enhancement of the viscosity of premelting films (c.f. [57]) over the bulk value. However, this appears to remain as an unsolved issue, with very recent high-profile studies being published [58]. Thus, here we use the viscosity of supercooled bulk water as reported in Supplementary Table 3. Changing the value of the viscosity in our model will not qualitatively change our results.

Data for all the parameters used in the Singe Gordon + Capillary Wave dynamical model may be found in Supplementary Table 3.

SUPPLEMENTARY NOTE 8: NUMERICAL SOLUTION OF THE GRADIENT DYNAMICS

Numerical computations of the dynamics of the interfaces predicted by our coupled partial differential equation model in Eqs. (50) and (51) (i.e. Eqs. (3) and (4) of the main text) are performed using a method of lines technique similar to that used in Ref. [59]. The method is extended to evolve the two interfaces (solid-liquid, and liquid-vapor), with coupling terms involving mass transfer and the two interface potentials naturally included. However, we evaluate the spatial derivatives in a different manner, which significantly increases the rate of numerical convergence. This was done because for the evolution of the solid-liquid interface, a pinning effect in the horizontal direction can occur if too few mesh points are used. Consequently, rather than using an extremely large number of points in the finite difference scheme used in [59], here we implement a periodic pseudospectral method.

The numerical method uses results from Ref. [60], discretising on a regular (periodic) grid and uses a band-limited interpolant derived using the discrete Fourier transform and its inverse to form the differentiation matrices which act in real space (see chapter 3 of [60] for details). The periodicity enabled by the premelting film avoids the need to evolve actual contact lines, in comparison to some of our previous work using pseudospectral discretisation [61, 62]. For the time stepping, the ode15s Matlab variable-step, variable-order solver is used [63]. Our numerical calculations are performed on the nondimensionalised version of the model equations. We find that choosing $\kappa_1^{-1} \approx 0.49$ nm and $3\eta/(\kappa_1\gamma_{\rm lv}) \approx 0.11$ ns as our units of length and time in the nondimensionalisation works well.

To explore the effectiveness of our model to at least qualitatively reproduce the phenomena observed in the experiments and to confirm the validity of the analytical predictions for the different behaviors in the different $(p_{\rm v}, T)$ regions of the phase diagram, we perform an extensive set of full numerical simulations, for a range of state points covering all the different growth regimes. Of course, the observed behavior also depends on the effective surface free energy $\omega_{\rm k}(h)$, which includes ice surface effects on the evolution of the interfaces, and on the initial conditions. A comprehensively large variety of initial conditions (i.e. the $t/\tau = 1$ profiles of the two interfaces) have also been trialled, especially for planar interfaces (at different separations, usually based on the heights corresponding to the α or β minima) with either small imperfections in the solid, or an initial perturbation of the liquid surface, or both. The results presented in the paper are drawn from the following three different initial condition types: Firstly, a planar solid-liquid surface with a Gaussian droplet shaped perturbation in the liquid-vapor interface, given by

$$L_{\rm lv} = d_{\rm B} + h_0 + A_{\rm f}\exp[-((x - x_{\rm L}/2)/x_{\rm wf})^2], \tag{81}$$

$$L_{\rm sl} = d_{\rm B}, \tag{82}$$

where h_0 is an initial separation (such as the height of the α minimum), $x_{\rm L}$ is the size of the periodic domain (taken as $x_{\rm L} = 2500\kappa_1^{-1}$) in all simulations presented here, $A_{\rm f} = 17\kappa_1^{-1}$ is the height of the Gaussian perturbation and $x_{\rm wf}$ is a measure of its width. We typically set $x_{\rm wf} = 450\kappa_1^{-1}$ for the results presented here.

The other two forms for the initial conditions are

$$L_{\rm sl} = d_{\rm B} \pm \frac{A_{\rm i}}{2} d_{\rm B}\left[\tanh\left(\frac{x - (x_{\rm L} - x_{\rm wi})/2}{10\kappa_1^{-1}}\right) - \tanh\left(\frac{x - (x_{\rm L} + x_{\rm wi})/2}{10\kappa_1^{-1}}\right)\right], \tag{83}$$

$$L_{\rm lv} = d_{\rm B} + h_0, \tag{84}$$

which corresponds to a planar liquid-vapor surface, together with an ice-liquid interface that has on it a small imperfection of hight $A_{\rm i}$ that is an integer multiple of the height of a single ice terrace, that protrudes either into or away from the liquid, and has width $x_{\rm wi}$. Values used in the work presented here are $\{A_{\rm i}, x_{\rm wi}\} = \{1, x_{\rm wi} = x_{\rm L}/16\}$ and $\{A_{\rm i}, x_{\rm wi}\} = \{10, x_{\rm wi} = 9x_{\rm L}/16\}$.

Fig. 4 and 5 of the main text displays snapshots from four typical simulations, and here we show snapshots from two additional simulations in Figs. 2–3. The full time evolutions of all six simulations can be seen in the movies included as supplementary material, named Movies S1–S6.

SUPPLEMENTARY REFERENCES

[1] Murata, K.-i., Asakawa, H., Nagashima, K., Furukawa, Y. & Sazaki, G. Thermodynamic origin of surface melting on ice crystals. *Proc. Natl. Acad. Sci. U.S.A.* **113**, E6741–E6748 (2016).

[2] Wagner, W. & Pruß, A. The IAPWS formulation 1995 for the thermodynamic properties of ordinary water substance for general and scientific use. *J. Phys. Chem. Ref. Data* **31**, 387–535 (2002).

[3] Feistel, R. & Wagner, W. A new equation of state for H2O ice Ih. *J. Phys. Chem. Ref. Data* **35**, 1021–1047 (2006).

[4] Murphy, D. M. & Koop, T. Review of the vapour pressures of ice and supercooled water for atmospheric applications. *Q. J. R. Meteorol. Soc.* **131**, 1539–1565 (2005).

[5] Pruppacher, H. R. & Klett, J. D. *Microphysics of Clouds and Precipitation* (Springer, Heidelberg, 2010).

[6] Hare, D. E. & Sorensen, C. M. The density of supercooled water. II. bulk samples cooled to the homogeneous nucleation limit. *J. Chem. Phys.* **87**, 4840–4845 (1987).

[7] Tanaka, M., Girard, G., Davis, R., Peuto, A. & Bignell, N. Recommended table for the density of water between 0°C and 40°C based on recent experimental reports. *Metrologia* **38**, 301–309 (2001).

[8] Fletcher, N. H. *The Chemical Physics of Ice* (Cambridge University Press, 1970). Cambridge Books Online.

[9] Taborek, P., Kleiman, R. N. & Bishop, D. J. Power-law behavior in the viscosity of supercooled liquids. *Phys. Rev. B* **34**, 1835–1840 (1986).

[10] MacDowell, L. G. & Müller, M. Observation of autophobic dewetting on polymer brushes from computer simulation. *J. Phys.: Condens. Matter* **17**, S3523–S3528 (2005).

[11] Grzelak, E. M. & Errington, J. R. Computation of interfacial properties via grand canonical transition matrix Monte Carlo simulation. *J. Chem. Phys.* **128**, 014710 (2008).

[12] Benet, J., Palanco, J. G., Sanz, E. & MacDowell, L. G. Disjoining pressure, healing distance, and film height dependent surface tension of thin wetting films. *J. Phys. Chem. C* **118**, 22079–22089 (2014).

[13] Hoyt, J. J., Olmsted, D., Jindal, S., Asta, M. & Karma, A. Method for computing short-range forces between solid-liquid interfaces driving grain boundary premelting. *Phys. Rev. E* **79**, 020601 (2009).

[14] Hickman, J. & Mishin, Y. Disjoining potential and grain boundary premelting in binary alloys. *Phys. Rev. B* **93**, 224108 (2016).

[15] Elbaum, M. & Schick, M. Application of the theory of dispersion forces to the surface melting of ice. *Phys. Rev. Lett.* **66**, 1713–1716 (1991).

[16] Llombart, P., Noya, E. G., Sibley, D. N., Archer, A. J. & MacDowell, L. G. Rounded layering transitions on the surface of ice. *Phys. Rev. Lett.* **124**, 065702 (2020).

[17] MacDowell, L. G. Surface van der waals forces in a nutshell. *J. Chem. Phys.* **150**, 081101 (2019).

[18] Parsegian, V. A. & Ninham, B. W. Temperature-dependent van der waals forces. *Biophys. J.* **10**, 664–674 (1970).

[19] Parsegian, V. A. *Van der Waals Forces* (Cambridge University Press, Cambridge, 2006).

[20] Chernov, A. A. & Mikheev, L. V. Wetting of solid surfaces by a structured simple liquid: Effect of fluctuations. *Phys. Rev. Lett.* **60**, 2488–2491 (1988).

[21] Evans, R. Density functionals in the theory of nonuniform fluids. In Henderson, D. (ed.) *Fundamentals of Inhomogenous Fluids*, chap. 3, 85–175 (Marcel Dekker, New York, 1992).

[22] Henderson, J. R. Wetting phenomena and the decay of correlations at fluid interfaces. *Phys. Rev. E* **50**, 4836–4846 (1994).

[23] Asakawa, H., Sazaki, G., Nagashima, K., Nakatsubo, S. & Furukawa, Y. Two types of quasi-liquid layers on ice crystals are formed kinetically. *Proc. Natl. Acad. Sci. U.S.A.* **113**, 1749–1753 (2016).

[24] Luengo, J. & MacDowell, L. *Van der Waals Forces at Ice Surfaces with Atmospheric Interest.* Master's thesis, Facultad de Ciencias (2020).

[25] Fiedler, J. *et al.* Full-spectrum high-resolution modeling of the dielectric function of water. *J. Phys. Chem. B* **124**, 3103–3113 (2020). PMID: 32208624.

[26] Benet, J., Llombart, P., Sanz, E. & MacDowell, L. G. Premelting-induced smoothening of the ice-vapor interface. *Phys. Rev. Lett.* **117**, 096101 (2016).

[27] Benet, J., Llombart, P., Sanz, E. & MacDowell, L. G. Structure and fluctuations of the premelted liquid film of ice at the triple point. *Mol. Phys.* **117**, 2846–2864 (2019).

[28] Llombart, P., Noya, E. G. & MacDowell, L. G. Surface phase transitions and crystal habits of ice in the atmosphere. *Sci. Adv.* **6** (2020).

[29] Grün, G., Mecke, K. & M., R. Thin–film flow influenced by thermal noise. *J. Stat. Phys.* **122**, 1261–1291 (2006).

[30] Davidovitch, B., Moro, E. & Stone, H. A. Spreading of viscous fluid drops on a solid substrate assisted by thermal fluctuations. *Phys. Rev. Lett.* **95**, 244505 (2005).

[31] Mecke, K. & Rauscher, M. On thermal fluctuations in thin film flow. *J. Phys.: Condens. Matter* **17**, S3515–S3522 (2005).

[32] Chui, S. T. & Weeks, J. D. Dynamics of the roughening transition. *Phys. Rev. Lett.* **40**, 733–736 (1978).

[33] Büttiker, M. & Landauer, R. Nucleation theory of overdamped soliton motion. *Phys. Rev. Lett.* **43**, 1453–1456 (1979).

[34] Saito, Y. Statics and dynamics of the roughening transition: A self-consistent calculation. In T., R. (ed.) *Ordering in Strongly Fluctuating Condensed Matter Systems*, 319–324 (Plenum, New York, 1980).

[35] Karma, A. Fluctuations in solidification. *Phys. Rev. E* **48**, 3441–3458 (1993).
[36] Bennett, C. H., Büttiker, M., Landauer, R. & Thomas, H. Kinematics of the forced and overdamped sine-gordon soliton gas. *J. Stat. Phys.* **24**, 419–442 (1981).
[37] Pototsky, A., Bestehorn, M., Merkt, D. & Thiele, U. Morphology changes in the evolution of liquid two-layer films. *J. Chem. Phys.* **122**, 224711 (2005).
[38] Noziéres, P. & Gallet, F. The roughening transition of crystal surfaces. i. static and dynamic renormalization theory, crystal shape and facet grwoth. *J. Phys.(Paris)* **48**, 353–367 (1987).
[39] Cuerno, R. & Moro, E. Dynamic renormalization group study of a generalized continuum model of crystalline surfaces. *Phys. Rev. E* **65**, 016110 (2001).
[40] Karma, A. & Plapp, M. Spiral surface growth without desorption. *Phys. Rev. Lett.* **81**, 4444–4447 (1998).
[41] Marconi, U. M. B. & Tarazona, P. Dynamic density functional theory of fluids. *J. Chem. Phys.* **110**, 8032–8044 (1999).
[42] Archer, A. J. & Rauscher, M. Dynamical density functional theory for interacting brownian particles: stochastic or deterministic? *J. Phys. A* **37**, 9325–9333 (2004).
[43] Prestipino, S. & Tosatti, E. Variational theory of preroughening. *Phys. Rev. B* **59**, 3108–3124 (1999).
[44] Moro, E. & Cuerno, R. Variational mean-field study of a continuum model of crystalline tensionless surfaces. *Phys. Rev. E* **63**, 036104 (2001).
[45] Dietrich, S., Nightingale, M. P. & Schick, M. Applicability of mean-field theory to wetting in binary-liquid mixtures. *Phys. Rev. B* **32**, 3182–3185 (1985).
[46] Lutsko, J. F. A dynamical theory of nucleation for colloids and macromolecules. *J. Chem. Phys.* **136**, 034509 (2012).
[47] Lutsko, J. F. How crystals form: A theory of nucleation pathways. *Sci. Adv.* **5**, eaav7399 (2019).
[48] Neshyba, S., Adams, J., Reed, K., Rowe, P. M. & Gladich, I. A quasi-liquid mediated continuum model of faceted ice dynamics. *J. Geophys. Res.: Atmos.* **121**, 14,035–14,055 (2016).
[49] Feistel, R. & Wagner, W. Sublimation pressure and sublimation enthalpy of h2o ice ih between 0 and 273.16 k. *Geochim. Cosmochim. Acta* **71**, 36–45 (2007).
[50] Batista, E. R., Ayotte, P., Bilić, A., Kay, B. D. & Jónsson, H. What determines the sticking probability of water molecules on ice? *Phys. Rev. Lett.* **95**, 223201 (2005).
[51] Neshyba, S., Nugent, E., Roeselova, M. & Jungwirth, P. Molecular dynamics study of ice-vapor interactions via the quasi-liquid layer. *J. Phys. Chem. C* **113**, 4597–4604 (2009).
[52] Pfalzgraff, W., Neshyba, S. & Roeselova, M. Comparative molecular dynamics study of vapor-exposed basal, prismatic, and pyramidal surfaces of ice. *J. Phys. Chem. A* **115**, 6184–6193 (2011).
[53] Llombart, P., Bergua, R. M., Noya, E. G. & MacDowell, L. G. Structure and water attachment rates of ice in the atmosphere: Role of nitrogen. *Phys. Chem. Chem. Phys* **21**, 19594–19611 (2019).
[54] Libbrecht, K. G. Towards a comprehensive model of snow crystal growth: 3. the correspondence between ice growth from water vapor and ice growth from liquid water. *Preprint at http://arxiv.org/abs/1407.0740* (2014).
[55] Büttiker, M. & Landauer, R. Nucleation theory of overdamped soliton motion. *Phys. Rev. A* **23**, 1397–1410 (1981).
[56] Pruppacher, H. R. Interpretation of experimentally determined growth rates of ice crystals in supercooled water. *J. Chem. Phys.* **47**, 1807–1813 (1967).
[57] Pramanik, S. & Wettlaufer, J. S. Confinement effects in premelting dynamics. *Phys. Rev. E* **96**, 052801 (2017).
[58] Canale, L. *et al.* Nanorheology of interfacial water during ice gliding. *Phys. Rev. X* **9**, 041025 (2019).
[59] Yin, H., Sibley, D. N., Thiele, U. & Archer, A. J. Films, layers, and droplets: The effect of near-wall fluid structure on spreading dynamics. *Phys. Rev. E* **95**, 023104 (2017).
[60] Trefethen, L. N. *Spectral Methods in MATLAB* (SIAM, 2000).
[61] Sibley, D. N., Savva, N. & Kalliadasis, S. Slip or not slip? a methodical examination of the interface formation model using two-dimensional droplet spreading on a horizontal planar substrate as a prototype system. *Phys. Fluids* **24**, 082105 (2012).
[62] Sibley, D. N., Nold, A. & Kalliadasis, S. The asymptotics of the moving contact line: cracking an old nut. *J. Fluid Mech.* **764**, 445–462 (2015).
[63] Shampine, L. F. & Reichelt, M. W. The MATLAB ODE Suite. *SIAM J. Sci. Comput.* **18**, 1–22 (1997).
