# Supplementary material for: How ice grows from premelting films and water droplets
Source: Nat Commun. 2021 Jan 11;12:239. doi: 10.1038/s41467-020-20318-6 (PMC7801427; doi:10.1038/s41467-020-20318-6)
Supplement: Supplementary file 3 — Description of Additional Supplementary Files [file 41467_2020_20318_MOESM3_ESM.pdf]

Description of Additional Supplementary Files

File Name: Supplementary Data 1
Description: GROMACS Configuration file of bulk ice with a random hydrogen network satisfying the ice rules.

File Name: Supplementary Movie 1
Description: Dynamics of a terrace in the activated growth region. Movie corresponding to Fig. 4 (a-e).

File Name: Supplementary Movie 2
Description: Dynamics of a liquid droplet quenched below the kinetic liquid-vapor coexistence line. Movie corresponding to Fig. 4 (f-j).

File Name: Supplementary Movie 3
Description: Growth of a liquid droplet above the kinetic liquid-vapor coexistence line. Movie corresponding to Fig. 5 (a-e).

File Name: Supplementary Movie 4
Description: Dynamics of a liquid droplet above the α to β transition line. Movie corresponding to Fig. 5 (f-j).

File Name: Supplementary Movie 5
Description: Dynamics of a liquid droplet above the kinetic liquid-vapor coexistence line. Movie corresponding to Supplementary Figure 2.

File Name: Supplementary Movie 6
Description: Dynamics of a liquid droplet past the kinetic spinodal line. Movie corresponding to Supplementary Figure 3.
